# Sero-epidemiological evaluation of *Plasmodium falciparum* malaria in Senegal

**DOI:** 10.1186/s12936-015-0789-x

**Published:** 2015-07-16

**Authors:** Khadime Sylla, Roger Clément Kouly Tine, Magatte Ndiaye, Doudou Sow, Aïssatou Sarr, Marie Louise Tshibola Mbuyi, Ibrahima Diouf, Amy Colé Lô, Annie Abiola, Mame Cheikh Seck, Mouhamadou Ndiaye, Aïda Sadikh Badiane, Jean Louis A N’Diaye, Daouda Ndiaye, Oumar Faye, Thérèse Dieng, Yémou Dieng, Oumar Ndir, Oumar Gaye, Babacar Faye

**Affiliations:** Service de Parasitologie-Mycologie, Faculté de Médecine, Pharmacie et Odontologie, Université Cheikh Anta Diop de Dakar, Dakar, Sénégal; Département de Parasitologie-Mycologie, Université des Sciences de la Santé, Libreville, Gabon

**Keywords:** Malaria, *Plasmodium falciparum*, Serology, Epidemiology, Senegal

## Abstract

**Background:**

In Senegal, a significant decrease of malaria transmission intensity has been noted the last years. Parasitaemia has become lower and, therefore, more difficult to detect by microscopy. In the context of submicroscopic parasitaemia, it has become relevant to rely on relevant malaria surveillance tools to better document malaria epidemiology in such settings. Serological markers have been proposed as an essential tool for malaria surveillance. This study aimed to evaluate the sero-epidemiological situation of *Plasmodium falciparum* malaria in two sentinel sites in Senegal.

**Methods:**

Cross-sectional surveys were carried out in Velingara (south Senegal) and Keur Soce (central Senegal) between September and October 2010. Children under 10 years old, living in these areas, were enrolled using two-level, random sampling methods. *P. falciparum* infection was diagnosed using microscopy. *P. falciparum* antibodies against circumsporozoite protein (CSP), apical membrane protein (AMA1) and merozoite surface protein 1__42_ (MSP1__42_) were measured by ELISA method. A stepwise logistic regression analysis was done to assess factors associated with *P. falciparum* antibodies carriage.

**Results:**

A total of 1,865 children under 10 years old were enrolled. The overall falciparum malaria prevalence was 4.99% with high prevalence in Velingara of 10.03% compared to Keur Soce of 0.3%. Symptomatic malaria cases (fever associated with parasitaemia) represented 17.37%. Seroprevalence of anti-AMA1, anti-MSP1__42_ and anti-CSP antibody was 38.12, 41.55 and 40.38%, respectively. The seroprevalence was more important in Velingara and increased with age, active malaria infection and area of residence.

**Conclusion:**

The use of serological markers can contribute to improved malaria surveillance in areas with declining malaria transmission. This study provided useful baseline information about the sero-epidemiological situation of malaria in Senegal and can contribute to the identification of malaria hot spots in order to concentrate intervention efforts.

Trial registration number: PACTR201305000551876 (http://www.pactr.org).

## Background

Despite increasing efforts to control malaria and many African countries reporting a decrease of malaria burden in recent years, the disease is still a major public health problem in many sub-Saharan African countries. According to the World Health Organization, there were an estimated 207 million malaria cases and 627,000 malaria deaths in the world in 2012. This situation justifies the need to strengthen malaria control strategies including: (1) clinical case management of malaria cases using rapid diagnostic test (RDTs) and artemisinin combination therapy (ACT); (2) universal coverage of long-lasting, insecticide-treated nets (LLINs); (3) indoor residual spraying (IRS); and, (4) intermittent preventive treatment [1–3]. In Senegal, the National Malaria Control Programme (NMCP) initiated the scaling-up of malaria control measures in 2005. Significant reduction of malaria morbidity has been noted these last years from 35.72% in 2001 to 5.62% in 2008 and 3.07% in 2009 [4]. Malaria parasitaemia has become lower and therefore more difficult to detect by microscopy with an increase in the proportion of individuals carrying submicroscopic malaria parasites [5]. This may induce some limitation in malaria surveillance using microscopy. To overcome this issue, there is a need to develop innovative malaria surveillance tools that are more sensitive and more reliable for better documentation of malaria epidemiology. For this purpose, serology is proposed as a reliable and sensitive tool to assess malaria epidemiology as well as malaria intervention impact on malaria burden and transmission [6–8]. Several *Plasmodium falciparum* antigens have been studied to assess malaria transmission and impact on the host immunity. To assess the level of malaria transmission, a pre-erythrocytic-stage antigen most commonly used is the circumsporozoite protein (CSP) with a short estimated half-life. Antibodies against this protein are correlated to transmission intensity and exposure duration, but not necessarily to plasmodial infections. This protein is labile and disappears quickly in the absence of exposure. *P. falciparum* erythrocytic-stage antigens, such as merozoite surface protein 1 (MSP) and apical membrane antigen (AMA1) with long half-lives, reflect the cumulative exposure to malaria and can be used as an indicator of the burden of malaria [6, 7].

The analysis of immune responses against pre-erythrocytic-stage antigen (CSP) and erythrocytic-stage antigens (MSP and AMA1) can contribute to assess malaria transmission and the impact on host immunity. This study was conducted to evaluate the sero-epidemiological situation of falciparum malaria using CSP, AMA1 and MSP1__42_ in the context of scaling anti-malarial interventions in Senegal.

## Methods

### Study area

This study was carried out in two health districts (Velingara and NDoffane) with a different endemicity level. Velingara health district is located in the southeastern part of Senegal, 500 km from the capital city of Dakar. In this district the study was conducted in Bonconto health post, which is headed by a nurse and has eight functional health huts staffed with community health workers, serving a population of 10,016 inhabitants. Ndoffane is located in the central part of Senegal, 200 km from Dakar. In this district the study was conducted in Lamarame health post. This health post is led by a nurse and comprises 49 functional health huts and serves a population of 20,000 inhabitants. In both study areas malaria transmission is seasonal, occurring during the rainy season (from July to November) with a peak in between October to November. *P. falciparum* is the most predominant parasite species. These two areas are part of NMCP sentinel sites. Malaria control strategies implemented by the NMCP in both sites were represented by the case management of uncomplicated malaria cases using rapid diagnostic tests (RDTs) and artemisinin combination therapy (ACT); intermittent preventive treatment in pregnant women; universal coverage of LLINs. The IRS is applied only in Velingara.

### Study design and population

A cross-sectional survey was conducted in Velingara and Keur Soce in September and October 2010, several years after the implementation of malaria control measures. Children under 10 years old, living in the area or who stayed at the site for at least 6 months and whose parents or legal representatives gave informed consent form approval, were enrolled in the study using a two-level, random sampling method. Subjects whose parents or legal representatives did not give informed consent were excluded from the study.

### Data collection method

An informed consent questionnaire was administered to collect individual data on socio-demographic (age, gender, weight, height, area of residence, bed net use). Weight and height were collected to determine nutritional status. In addition, axillary temperature was measured.

## Laboratory methods

### Parasitological assessment

For each enrolled participant, three drops of blood were collected for thick and thin smear tests for the detection of malaria prevalence using microscopy. Slides were stained for 15 min with a 10% Giemsa solution. Parasite density was evaluated by counting the number of asexual parasites per 200 white blood cells (WBCs) and estimated by number of parasite per μl using the following formula: number of parasites × 8,000/200 assuming a WBC count 8,000 cells/µl. Thick and thin smears were considered as negative after 100-field microscopic reading without any parasites being detected.

## Serological assessment

### Malaria antigens

Apical membrane antigen (AMA1) was from the *Pichia pastoris* expressed ectodomain of *P. falciparum* FVO strain comprised amino acids 25–545 [9] (Donated by Dr Daniel Dodo, Noguchi Memorial Institute for Medical Research, University of Ghana, Legon, Ghana).

MSP1__42_ protein was from the C-terminal MSP1__42_ amino acid sequence of the Uganda-Palo Alto (FUP) *P. falciparum* isolate (GenBank Accession No. M37213) expressed in *Escherichia coli* (*Ec*) system. The recombinant protein, EcMSP1__42_-FUP (Uganda-Palo Alto strain), represents the 33 kDa fragment from the 3D7 *P. falciparum* variant and the E-K-NG point mutations identified in the 19 kDa fragment within the MSP1__42_ native molecule [10].

CSP was a full-length protein expressed in an *Escherichia coli* system containing amino-acids Leu^19^ to Ser^411^ (Indian isolate, GenBankTM No: AAN87606) [11].

MSP1__42_ and CPS were donated by Dr Patrick Duffy and Dr Richard Shimp from NIH/NIAID (National Institutes of Health/National Institute of Allergy and Infectious Diseases).

### Enzyme-linked immunosorbent assay (ELISA)

Three drops of blood were collected onto Whatman 3MM filter paper, which was sealed and stored dry with desiccant at room temperature. Reconstituted sera were obtained from filter paper bloods spots described previously [12, 13]. Sera were tested for anti-MSP1__42_ IgG antibodies, anti-AMA1 IgG antibodies and anti-CSP antibodies by indirect ELISA. Samples were also tested on freeze thawed *P. falciparum* schizont extract (concentration of 1 × 108/ml), which was coated onto ELISA plates at 1/500.

Briefly, 96 well ELISA plates were coated with 100 µl/well of 0.1 μl/well of MSP1__42_ and CSP antigens and 0.026 μl/well of AMA1 in coating buffer (1.59 g Na_2_CO3, 2.93 g NaHCO3, 1 liter distilled water, pH 9.4). The plates were incubated overnight at 4°C. After incubation, plates were washed at three times using PBS (5.7 g NaH_2_PO_4_, 16.7 g Na_2_HPO_4_, 85 g NaCl in 1 l distilled water) plus 0.05% Tween 20 (PBS/T) and blocked with 1% (w/v) skimmed milk power in PBS/T for 1 h at 37°C. Eluates were removed from 4°C just before use. After three more washes, eluates were diluted at a ration 1/100 in PBS/T and added 200 µl in duplicate in a well plate.

For each plate three types of control were used: deep well without serum but with a second antibody to measure the non-specific binding, pool of sera from patients with *P. falciparum* malaria (positive control) and pool of sera from non-infected subjects (negative control) from Copenhagen. Three washes were performed before incubation for 1 h at 37° with secondary antibody (100 µl of horseradish peroxidase-conjugated rabbit anti-human IgG, SouthernBiotech ^®^). After incubation for 1 h at 37°C, plates were developed with TMB/E (Upstate^®^, Chemicon^®^ et Linco^®^, Millipore) as substrate for 30 min at room temperature in the absence of light and the reaction was stopped by the addition of 50 µl/well of 2 M H_2_SO_4_. Optical density was read at 450 nm against a 620 nm with an ELISA TECAN SUNRISE reader.

### Haematological assessment

One drop of blood was collected from all participants for haemoglobin (Hb) level measurement using Hemo-Cue machine (HemoCue^®^ Hb 301). Anaemia was defined as Hb concentration below 11 g/dl.

### Statistical methods

After data collection, date entry work was performed using Excel software. Thereafter, analysis was carried out using Stata software version IC 12 software.

For serological assessment, the optical density was obtained by subtracting the average OD (Optical density) of duplicate wells from that of the corresponding blank wells. Values were converted into arbitrary units (AUs), as follows [14]:

$$AU\; = 100 \times \left[ {\frac{Ln(OD\;test\;sample) - Ln(OD\;negative\;control)}{Ln(OD\;positive\;control) - Ln(OD\;negative\;control)}} \right]$$

To assess the nutritional status, data were transferred into Epi Info 3.04 d. The *Z*-scores for weight-for-age (underweight) and height-forage (stunting) were derived using Epinut Anthropometry. Children with *Z*-scores below—2 standard deviation (SD) of the National Centre for Health Statistic (NCHS), United States reference population median were considered to be malnourished.

Quantitative variables were described in terms of means, SD. Inter-group comparisons were done using ANOVA test or Student t test where appropriate, otherwise non-parametric tests such as Mann–Whitney or Kruskal–Wallis were used.

For descriptive data, percentage was used to each outcome. Antibodies seroprevalence was calculated and expressed by percentage with their 95% confidence intervals. Proportions were compared using Chi square test or the Fisher exact test (univariate analysis). A stepwise logistic regression analysis was done to assess factors associated with *P. falciparum* antibodies carriage. Significance level of the different tests was set at 5%.

### Ethical considerations

The study was nested into a cluster-randomized trial [15] which had been approved by the Senegalese National Ethical Committee (*Conseil National d’Ethique et de Recherche en Santé)* and registered at the Pan African Clinical Trial Registry: registration number: PACTR201305000551876. In the field, individual informed consent was required prior to each participant enrolment. Community sensitization was done prior to the study to explain the planned investigations.

## Results

### Study participant characteristics

A total of 1,865 participants were studied (866 from Velingara and 999 from Keur Soce). The mean age of the study population was 4.24 ± 2 years. The study population was mainly represented by children under 5 years old (53.62%). A proportion of 7.83% were less than 1 year old. Children over 5 years represented 38.55%. The sex ratio was 1.03. The mean Hb level was 9.93 ± 3.3 g/dl and was lower in Velingara (8.5 ± 3.4 g/dl). The overall prevalence of anaemia (Hb <11 g/dl) was 72.39% with a higher proportion in Keur Soce (77.14%) than in Velingara 68.27% (p < 10^−3^).

The prevalence of stunting, underweight and wasting was respectively 35.44, 26.65 and 10.51%. Stunting was more frequent in Velingara population while underweight and wasting were higher in Keur Soce. Study participants characteristics are summarized in Table 1.

**Table 1:** Baseline characteristics of subjects

	Total (N = 1,865)	Velingara (N = 866)	Keur Soce (N = 999)
Mean age (year)	4.24 ± 2	4.5 ± 2.7	3.87 ± 1.9
Age group (year)			
<1	146 (7.83%)	86 (58.9%)	60 (41.01%)
1–4	1000 (53.62%)	344 (34.4%)	656 (65.67%)
5–10	719 (38.55%)	436 (60.64%)	283 (28.33%)
Gender			
Female	917 (49.17%)	443 (51.15%)	474 (47.45%)
Male	948 (50.83%)	423 (48.85%)	525 (52.55%)
Hb mean (g/dl)	9.93 ± 3.3	8.5 ± 3.4	10.16 ± 4.2
Anemia (Hb <11 g/dl)			
Yes	1350 (72.39%)	668 (77.14%)	682 (68.27%)
No	515 (27.61%)	198 (22.86%)	317 (31.73%)
Stunting			
Yes	661 (35.44%)	379 (43.76%)	282 (28.23%)
No	1204 (64.56%)	487 (56.24%)	717 (71.77%)
Underweight			
Yes	497 (26.65%)	214 (24.71%)	283 (28.33%)
No	1368 (73.35%)	652 (75.29%)	716 (71.67%)
Wasting			
Yes	196 (10.51%)	47 (5.43%)	149 (14.91%)
No	1669 (89.49%)	819 (94.57%)	850 (85.09%)

### Malariaometric indices

Overall, the coverage rate of bed net use was 82.84%. The coverage rate of bed net was more higher in Velingara (95.41%) while in Keur Soce it was 72.07%. The proportion of subjects with fever (axillary temperature ≥37.5°C) at the time of survey was 20.4 and 14.71% in Velingara and Keur Soce, respectively. Overall *Plasmodium* *falciparum* malaria prevalence was 4.99% [95% CI (4.02–6.1)]. Malaria prevalence was higher in Velingara at 10.03% [95% CI (8.3–12.7)] (90/866) than in Keur Soce, where it was 0.3% [95% CI (0.06–0.8)] (3/999). Among children with falciparum malaria, 17.37% had fever providing an odds ratio at 1.81 [95% CI (1.13–2.91)] (Table 2).

**Table 2:** Paludometrics indices

	Total (N = 1865)	Velingara (N = 766)	Keur Soce (N = 999)
Bets net use			
Yes	1.545 (82.84)	852 (95.41%)	720 (72.07%)
No	320 (17.16)	41 (4.59%)	279 (27.93%)
Fever			
Yes	324 (17.37%)	177 (20.4%)	147 (14.71%)
No	1541 (82.63%)	689 (79.56%)	852 (85.29%)
*Pf* malaria prevelance	93 (4.99%)	90 (10.3%)	3 (0.3%)

The mean production of anti-AMA1 antibody was 16.04 AU and varied from 16.59 AU in Velingara to 15.57 AU in Keur Soce. No significance difference was noted between the two sites (p = 0.57). MSP1__42_ and CSP antibodies was 15.17 AU and 29.25 UA, respectively; both were higher in Velingara’s population (p < 10^−3^). Antibodies production was significantly higher among children with *P. falciparum* infection compared to non-infected children (Table 3).

**Table 3:** Mean production of anti-AMA1, anti-MSP1__42_ and anti-CSP antibodies

	Total (N = 1,865)	Velingara (N = 866)		Keur Soce (N = 999)		p value
	General mean (AU)	Mean (AU)	IC (95%)	Mean (AU)	IC (95%)	
AMA1	16.04	16.59	14.3–18.8	15.57	12.8–18.3	0.57
MSP1__42_	15.17	21.7	19.1–24.3	9.5	8.2–10.7	<10^−3^
CSP	29.25	48.2	40.1–56.4	12.7	11.5–13.9	<10^−3^

**Figure 1:**
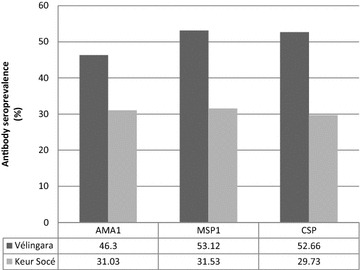
Seroprevalence of anti-CSP, anti-AMA1 and anti-MSP1__42_ antibody in Velingara and Keur Soce site. Seroprevalence of IgG antibody to different malaria antigens (AMA1, MSP1__42,_ and CSP). AMA1 (Apical Membrane Protein), MSP1__42_ (Merozoite Surface Protein) and CSP (Circumsporozoite Protein). *Black* Velingara site (southern part of Senegal) and *grey* Keur Soce site (central part of Senegal). χ^2^ test was done to compare proportions of antibody responders between two sites. Proportion of seropositive was more important in Velingara site compared to Keur Soce site (p < 10^−3^).

The overall seroprevalence rate of anti-AMA1, anti-MSP1__42_ and anti-CSP antibodies was at 38.12% [95% CI (35.37–41.03)], 41.55% [95% CI (38.68–44.58)] and 40.38% [95% CI (37.54–43.36)], respectively. The seroprevalence of these antibodies was higher in Velingara compared to Keur Soce (p < 10^−3^) (Figure 1).

Multivariate logistic regression analyses revealed that seroprevalence increased with age, active malaria infection and area of residence. In children over 5 years old, the seroprevalence of anti-AMA1 antibody was 46.1% [ORa = 1.18; IC (0.8–1.73), p value = 0.43] and this was more important among female children (38.7%), subjects with stunting (37.97%) and subjects without anaemia (43.88%). Seroprevalence of anti-AMA1 antibody was higher in subjects with *P. falciparum* infection 40.86% [ORa = 1.13; IC (0.74–1.72); p value = 0.56] and in subjects living in Velingara 46.3% [ORa = 1.85; IC (1.58–2.32); p < 10^−3^]. The seroprevalence of anti-MSP1__42_ antibody was 37.3% [ORa = 1.05; IC (0.73–1.51); p value = 0.8] in children under 5 years old while it was more important in children over 5 years old 47.57% [ORa = 1.28; IC (0.87–1.87), p value = 0.43].

For children with malaria infection, the seroprevalence of anti-MSP1__42_ antibody was 52.69% [ORa = 1.01; IC (0.65–1.55); p value = 0.95]. For children over 5 years, the seroprevalence of anti-CSP antibody was 43.53% [ORa = 1.08; IC (0.75–1.56); p = 0.66] compared to other children. Anti-CSP antibody was more important in female children (41%), children with stunting 43.72% [ORa = 1.09; IC (0.85–1.4); p = 0.45] and children with anaemia (40.44%). The prevalence of anti-CSP antibody was associated with active malaria infection. Prevalence of anti-CSP antibody was more important in children with malaria infection 46.24% [ORa = 1.2; IC (0.78–1.84); p = 0.4]. The prevalence of anti-CSP antibody was more important in Velingara 52.66% [ORa = 2.63; IC (2.13–3.24); p < 10^−3^] compared to Keur Soce (29.73%). There is no correlation between seroprevalence of antibody and sex (Tables 4, 5, 6).

**Table 4:** Factors influencing the seroprevalence of anti-AMA1 antibodies

	Number (%)	OR (95% CI)	ORa (95% CI)	p value
Age group (year)				
<1	57 (39.1%)	1	1	
1–4	322 (32.2%)	0.74 (0.5–1.06)	0.82 (0.56–1.18)	0.28
5–10	332 (46.1%)	1.34 (0.93–1.92)	1.18 (0.8–1.73)	0.43
Gender				
Female	355 (38.7%)	1	1	
Male	356 (37.5%)	0.95 (0.79–1.14)	0.97 (0.81–1.18)	0.82
Nutritionnel status				
Stunting	251 (37.97%)	0.99 (0.81–1.2)	0.94 (0.83–1.21)	0.62
Underweight	182 (36.62)	0.92 (0.74–1.13)	0.97 (0.87–1.2)	0.79
Wasting	62 (31.63%)	0.73 (0.53–0.99)	0.81 (0.57–1.15)	0.25
Anemia				
No	226 (43.88%)	1	1	
Yes	485 (35.93%)	0.72 (0.58–0.88)	0.72 (0.57–0.92)	0.005
Malaria parasite				
No	673 (37.98%)	1	1	
Yes	38 (40.86)	1.23 (0.74–1.72)	1.13 (0.74–1.72)	0.56
Residence area				
Keur Soce	310 (31.03%)	1	1	
Velingara	401 (46.30%)	1.92 (1.58–2.32)	1.85 (1.58–2.32)	<10^−3^

**Table 5:** Factors influencing the seroprevalence of anti-MSP1__42_ antibodies

	Number (%)	OR (95% CI)	ORa (95% CI)	p value
Age group (year)				
<1	60 (41.1%)	1	1	
1–4	373 (37.3%)	0.85 (0.59–61.21)	1.05 (0.73–1.51)	0.8
5–10	342 (47.57%)	1.3 (0.96–1.86)	1.28 (0.87–1.87)	0.19
Gender				
Female	385 (41.98%)	1	1	
Male	390 (41.14%)	0.96 (0.8–1.16)	0.98 (0.82–1.18)	0.87
Nutritional status				
Stunting	286 (43.27%)	1.15 (0.92–1.45)	0.97 (0.75–1.24)	0.8
Underweight	205 (41.25)	0.93 (0.71–1.2)	0.93 (0.82–1.43)	0.59
Retard staturo-pondéral	75 (38.27%)	0.89 (0.64–1.24)	0.93 (0.67–1.29)	0.68
Anemia				
No	213 (41.36%)	1	1	
Yes	562 (41.63%)	1.02 (0.82–1.24)	0.99 (0.78–1.23)	0.86
Malaria parasite				
No	726 (40.97%)	1	1	
Yes	49 (52.69%)	1.6 (1.05–2.43)	1.01 (0.65–1.55)	0.95
Residence area				
Keur Soce	315 (31.53%)	1	1	
Velingara	460 (53.12%)	2.46 (2.04–2.97)	2.4 (1.95–2.95)	<10^−3^

**Table 6:** Factors influencing the seroprevalence of anti-CSP antibodies

	Number (%)	OR (95% CI)	ORa (95% CI)	p value
Age group (year)				
<1	62 (42.47%)	1	1	
1–4	378 (37.8%)	0.82 (0.57–1.17)	0.85 (0.59–1.21)	0.38
5–10	313 (43.53%)	1.04 (0.73–1.5)	1.08 (0.75–1.56)	0.66
Gender				
Female	376 (41%)	1	1	
Male	377 (39.77%)	0.95 (0.78–1.14)	0.98 (0.81–1.19)	0.86
Nutritional status				
Stunting	289 (43.72%)	1.24 (1.02–1.5)	1.09 (0.85–1.4)	0.45
Underweight	191 (38.43%)	0.89 (0.72–1.1)	0.88 (0.64–1.09)	0.19
Wasting	55 (28.06%)	0.54 (0.4 0.75)	0.61 (0.43–0.86)	0.005
Anemia				
No	207 (40.19%)	1	1	
Yes	546 (40.44%)	1.01 (0.82–1.24)	0.87 (0.69–1.1)	0.25
Malaria parasite				
No	710 (40.07%)	1	1	
Yes	43 (46.24%)	1.28 (0.085–1.95)	1.2 (0.78–1.84)	0.4
Residence area				
Keur Soce	297 (29.73%)	1	1	
Velingara	456 (52.66%)	2.63 (2.17–3.17)	2.63 (2.13–3.24)	<10^−3^

## Discussion

In Senegal malaria is still a leading cause of morbidity and mortality. These last years, the combination of malaria control measures has helped to reduce malaria burden. This has led the country to outline a vision of malaria elimination. To address this issue, new approaches are fundamental for a better characterization of malaria epidemiology in the areas of reduced transmission. Serology has been proposed as a sensitive and reliable tool for malaria epidemiology assessment [16–19].

This study was conducted to assess the sero-epidemiological situation of malaria in two sentinel sites with different epidemiological profiles in Senegal. The study showed a low prevalence of malaria parasitaemia, although *P. falciparum* carriage was significantly higher in the southern part of the country (Velingara). However, anaemia remained high in both sites. These data are consistent with results from national malaria indicator surveys conducted 2008–2009, which showed similar patterns in terms of *P. falciparum* carriage and anaemia prevalence [20]. The national survey conducted in 2012 and 2013 showed an overall prevalence of *P. falciparum* at 2.8% with disparities between the southern part (9.3%) and the central part (2.2%) of the country [21]. The difference in malaria prevalence between the two areas demonstrates once again the heterogeneity of malaria transmission in Senegal. This was demonstrated in Gambia in 2008 and 2009 [22].

Similar results were found in 2005 with the heterogeneity of malaria in the east and west of Cambodia [23]. Despite the low prevalence of *P. falciparum*, anaemia was closely associated with malaria parasitaemia. Other studies demonstrated that the main factors influencing anaemia occurrence in the central and the southern parts of the country are mainly represented by *P. falciparum* carriage, malnutrition, sickle cell traits and alpha-thalassaemia [24].

The overall seroprevalence of AMA1, MSP1__42_ and CSP antibodies was 38.12, 41.55 and 40.38%, respectively. Significant difference between the two areas was observed with a higher seroprevalence in the southern part (Velingara). Although the serological assessment confirmed malaria heterogeneity as shown by microscopy. Proportion of *P. falciparum* carriage was significantly lower than antibodies level. These findings are in accordance with what were observed in Madagascar [6]. In Tanzania, similar results were noted with a high seroprevalence of antibodies against AMA1 (40.7%) and MSP1 (64.1%) [19]. Similar results were found in Ghana with high seroprevalence of AMA1 and CSP antibodies [25]. A high prevalence of PfMSP1 and PfAMA1 antibodies was found in Indonesia whatever the area and the season [26].

These results show that serology could be a good indicator for malaria surveillance. 5% of the total study participant was found positive by microscopy while antibodies excretion increased by at least sixfolds. The study demonstrated that using microscopy alone for malaria surveillance could underestimate malaria burden, particularly in areas with reduced malaria transmission. These data are confirmed by other studies [6, 19, 23, 27].

The study showed that seroprevalence of AMA1, MSP1__42_ and CSP antibodies increased with age, *P. falciparum* carriage and the area of residence. Similar results were found in Vanuatu in 2008 and in northern Peru between 2008 and 2010 [28, 29]. In 2002, a study in Ghana showed that the level of antibody was higher among older subjects [30]. This was also demonstrated in The Gambia and Senegal in 2002 [22, 31]. In children with *P. falciparum* infection, the seroprevalence of antibodies is higher compared to those without *P. falciparum* infection. The association between malaria and level of antibody (anti-AMA1, anti-MSP1__42_ and anti-CSP) has been demonstrated by several immuno-epidemiological studies [32–39]. Comparing both sites, the seroprevalence of antibodies is higher in Velingara than in Keur Soce. This may be due to the fact that malaria transmission is most intense in southern Senegal. The variation of malaria between areas has been demonstrated [22].

Gender, Hb level and nutritional status did not play a role in antibody production. This was demonstrated in 2002 in Senegalese preschool children when assessing the immunological consequences of intermittent preventive treatment [30].

Serological markers can be a useful tool for malaria epidemiology characterization particularly in areas with a decrease of malaria and can even contribute to the identification of malaria hot spots in order to concentrate intervention efforts. Others studies suggest that sero-epidemiological analysis will be useful tool in assessing short-term changes in exposure and malaria transmission in area with a low or seasonal transmission. It was demonstrated in Ghana, Kenya and Indonesia [40, 41].

### Study limitation

The age of the study population being limited to 10 years constituted a study limitation. To better document the changing profile of malaria epidemiology, it would be relevant to extend the study to the other age groups in order to characterize the burden of the disease in the study areas.

## Conclusion

Serological markers can be used as complementary tools for malaria survey in areas with a declining pattern of malaria in Senegal. This study provided useful baseline information about the sero-epidemiological situation of malaria in Senegal and can contribute to the identification of malaria hot spots in order to concentrate intervention efforts.
